# Supplementary material for: DNA methylation as a pharmacodynamic marker of glucocorticoid response and glioma survival
Source: Nat Commun. 2022 Sep 20;13:5505. doi: 10.1038/s41467-022-33215-x (PMC9486797; doi:10.1038/s41467-022-33215-x)
Supplement: Supplementary file 1 — Supplementary Information [file 41467_2022_33215_MOESM1_ESM.pdf]

Supplementary Information

Supplementary Figures:

Supplementary Figure 1. DEX neutrophil specific CpG methylation sites co-localize with H3K4me1 enhancer marks in blood monocytes

Supplementary Figure 2. NDMI scores associated with prednisone use in controls

Supplementary Figure 3. Post dexamethasone NDMI scores are identical to pre-exposure scores in subjects with 7 or more days of drug cessation

Supplementary Figure 4. Increase in NDMI score during DEX treatment is inversely associated with pre-drug NDMI status

Supplementary Figure 5. Simulation studies of NDMI and neutrophil proportions

Supplementary Figure 6. Bland Altman analysis of CD4 T cell count by FCM and methylation deconvolution

Supplementary Figure 7. A series of boxplots showing the relationship between the Dirichlet concentration parameter on the variability of cellular proportions

Supplementary Figure 8. FACS gating strategy

Supplementary Tables:

Supplementary Table 1. Clinical and demographic characteristics of study groups

Supplementary Table 2. FCM estimation of CD4 T cells and mMDSCs

Supplementary Table 3. Training dataset (N=135) blood leukocyte parameters and Dexamethasone status

Supplementary Table 4. Neutrophil specific loci CpG loci and gene characteristics, 28 NDMI CpG sites

Supplementary Table 5. Lists of explanatory variables and coefficients in three DEX predictors

Supplementary Table 6. FACS isolation of putative mMDSCs

Supplementary Table 7. Reproducibility of immune parameters: coefficients of variation of DEX predictors and 6 cell proportions

Supplementary Table 8. Associations of NDMI score with demographic and medication history in non-glioma controls (n=454)

Supplementary Table 9. Immune Profile Comparisons by Glucocorticoid Use at Blood Draw, Adult Glioma Study Controls

Supplementary Table 10. Isolated Neutrophil NDMI and Whole Blood NDMI Scores for Samples from Dexamethasone Exposed Patients

Supplementary Table 11. Cox multivariate model of NDMI and glioma survival in 74 UCSF Adult Glioma Study patients with extent of resection (EOR) and tumor volume available (both EOR and pre-operative tumor volume added to the model)

Supplementary Table 12. Cox multivariate model of NDMI and glioma survival in 74 UCSF Adult Glioma Study patients with extent of resection (EOR) and tumor volume available (only EOR added to the model)

Supplementary Table 13. Antibodies for FACS Isolation of Neutrophils

Supplementary References

Supplementary Figure 1. Output of the eFORGE program (all histone marks) using the 28 CpG sites contained in the NDMI algorithm (see Supplementary Table 4). The analysis shows the enhancer H3K4me1 chromatin mark is overrepresented in primary human monocytes (red data point FDR q<0.01). Note: primary neutrophils are not represented in the eFORGE database. Source data are provided as a Source Data file.

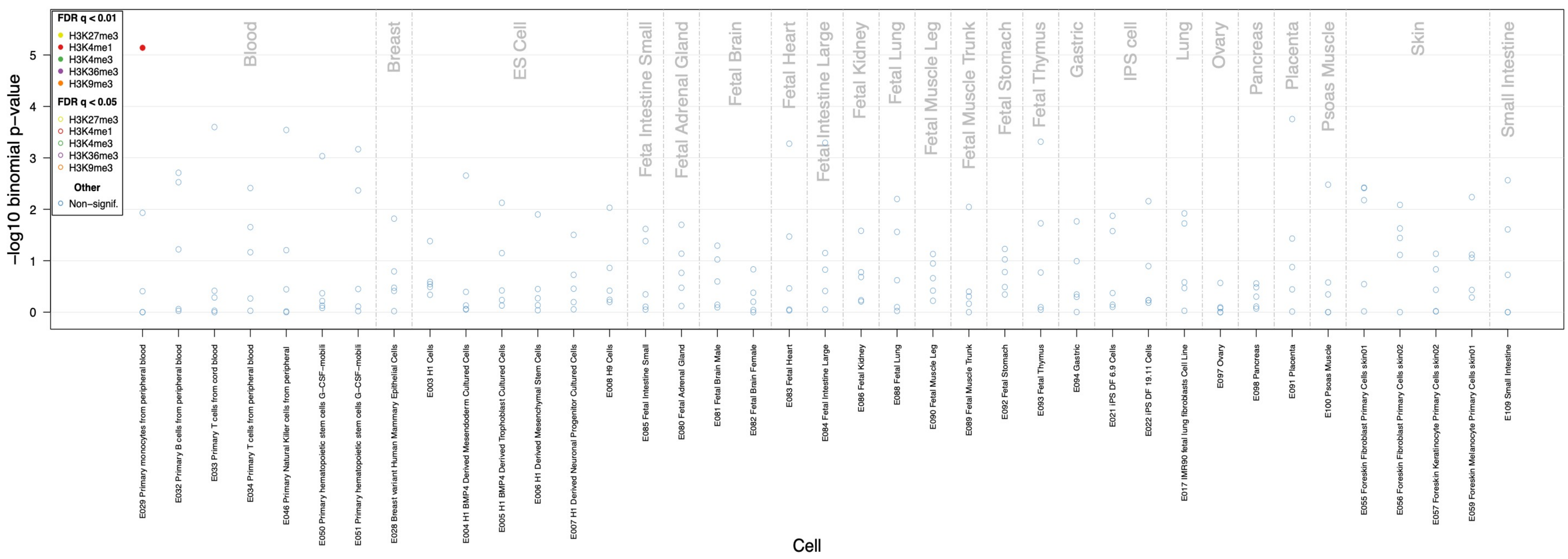

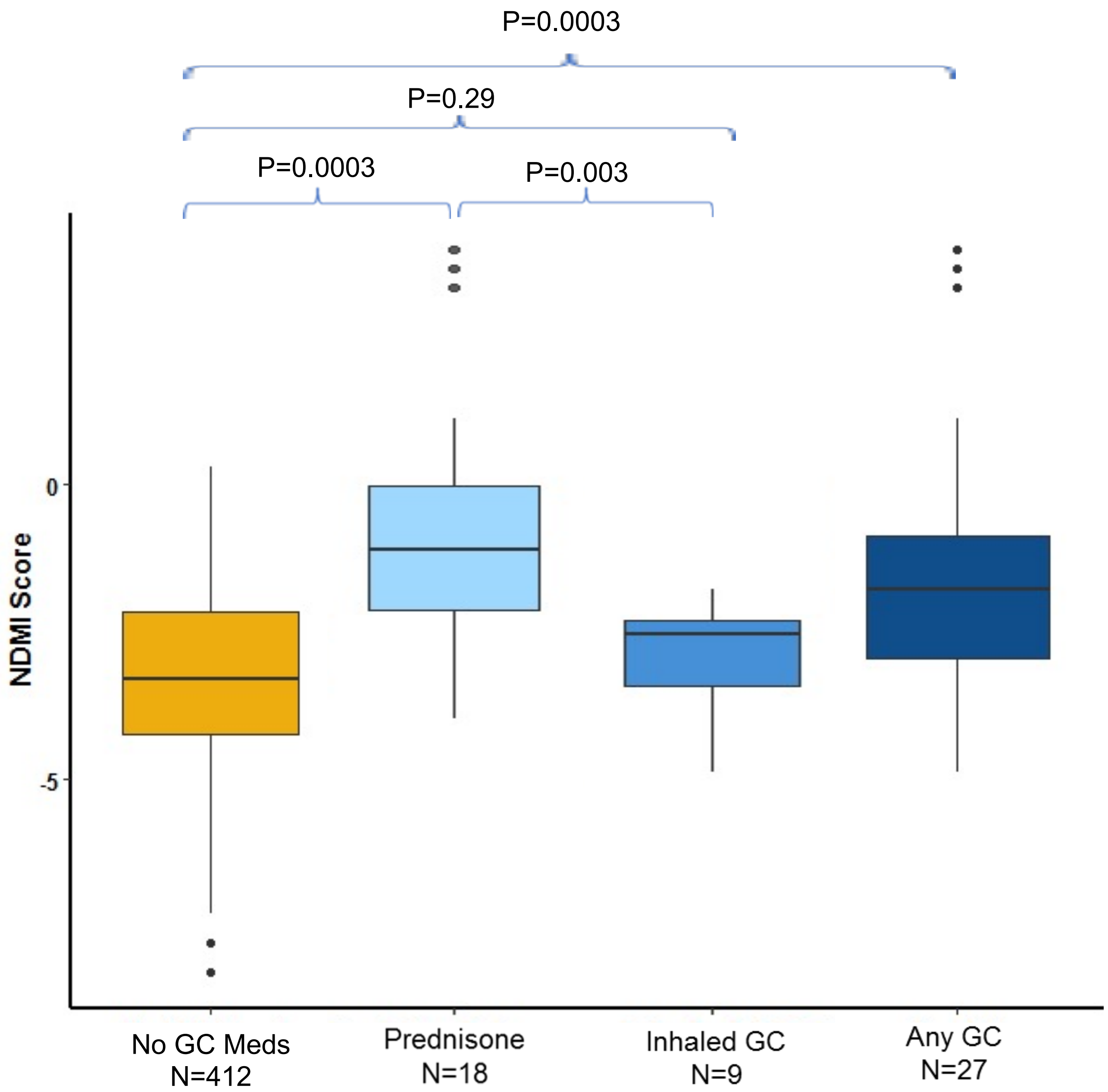

Supplementary Figure 2. History of prednisone and oral glucocorticoid medications in AGS control subjects and NDMI scores (see Supplementary Table 8). Controls with no prednisone or oral glucocorticoid medication history are compared with subjects reporting current use of these medications. The center lines within the boxes represent the median NDMI score and the upper and lower bounds of the boxes represent the IQR. The lines outside the boxes represent values within 1.5 x the upper and lower IQR, and the black dots represent outliers outside of this range. The p-values were calculated from 2-sided t-tests. Source data are provided as a Source Data file.

Supplementary Figure 3. Bivariate plot of presurgery and postsurgery NDMI scores (n=15 samples). No presurgery glioma subjects were exposed to DEX. After surgery all patients received a course of DEX and donated a blood sample at different times post exposure. A line of identity is plotted that represents identical presurgery and postsurgery scores. Subjects donating blood after 7 days following a course of DEX had NDMI scores falling on the line of identity. Source data are provided as a Source Data file.

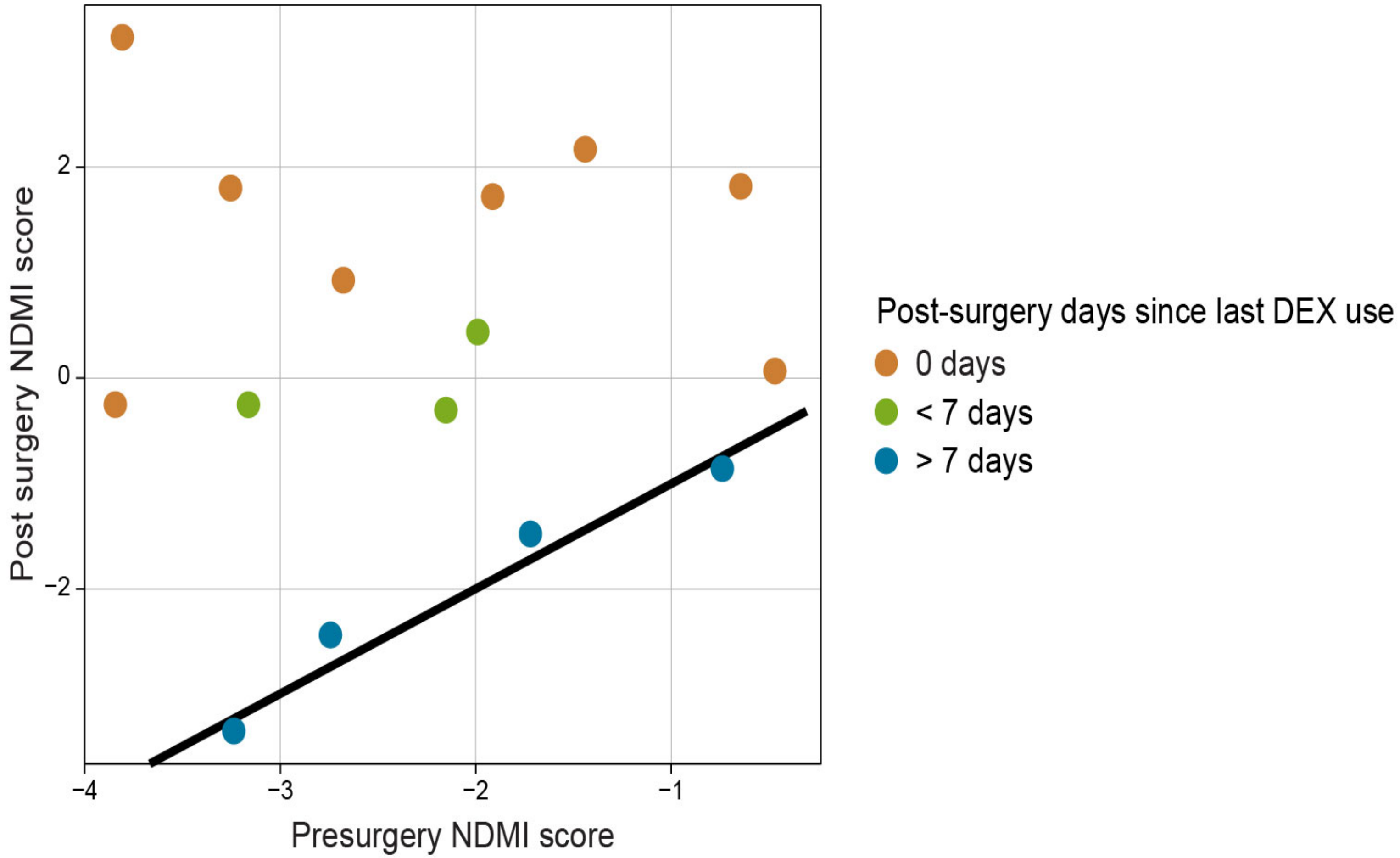

Supplementary Figure 4. Increase in NDMI scores following DEX treatment is plotted on the Y-axis in N=31 glioma subjects. The presurgery NDMI score is plotted on the X-axis. All subjects were DEX non-exposed prior to surgery and provided blood samples postsurgery samples while taking DEX. Increase in NDMI was inversely related to the presurgery NDMI score (Simple linear regression, r2=0.23; P=0.008). Source data are provided as a Source Data file.

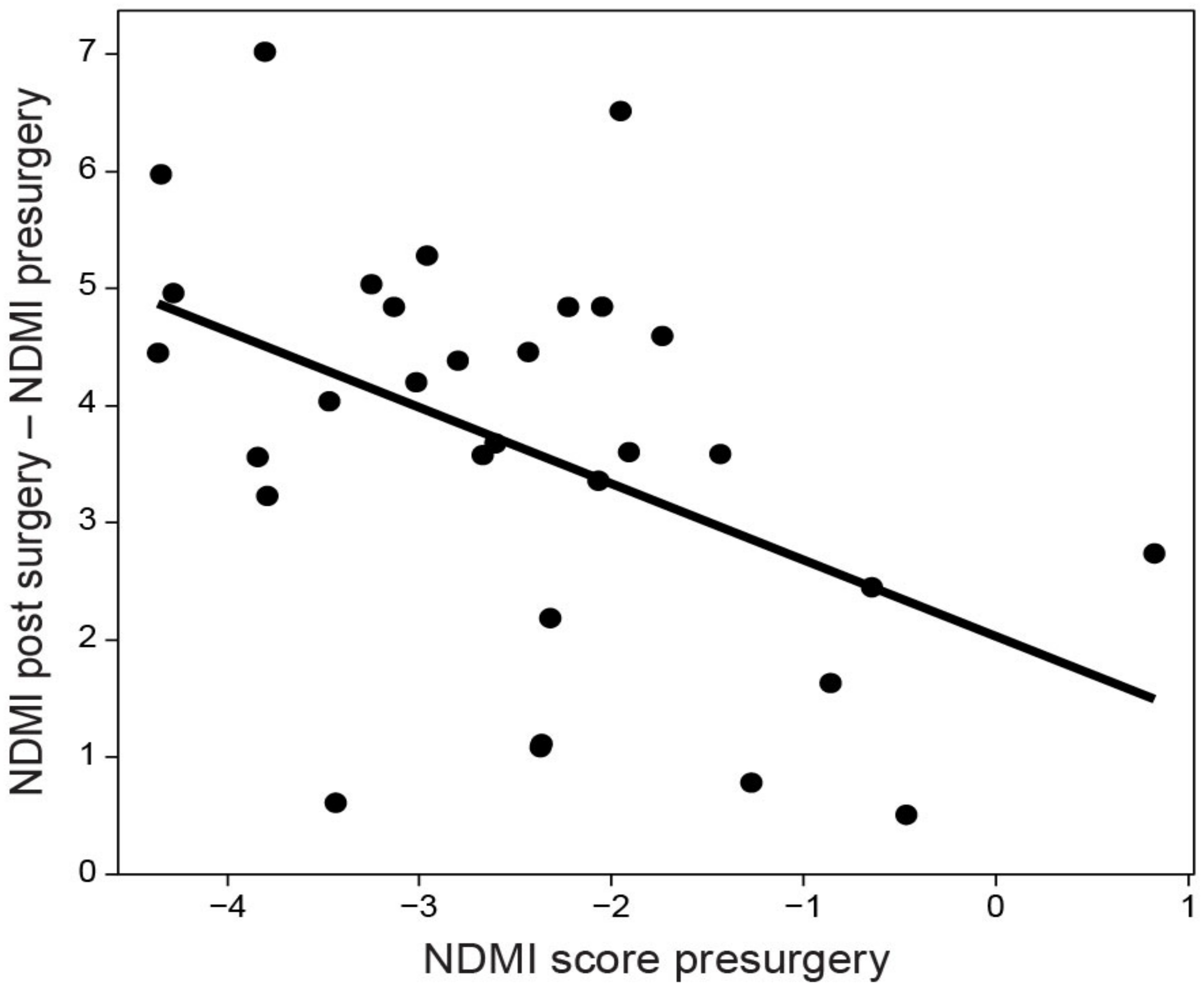

Supplementary Figure 5. Simulation studies modeling the power to detect DEX exposure at varying proportions of neutrophils and concentration parameter levels. Source data are provided as a Source Data filePanels A-D represent assumed sample sizes (N) of 20, 40, 60, and 80, respectively.

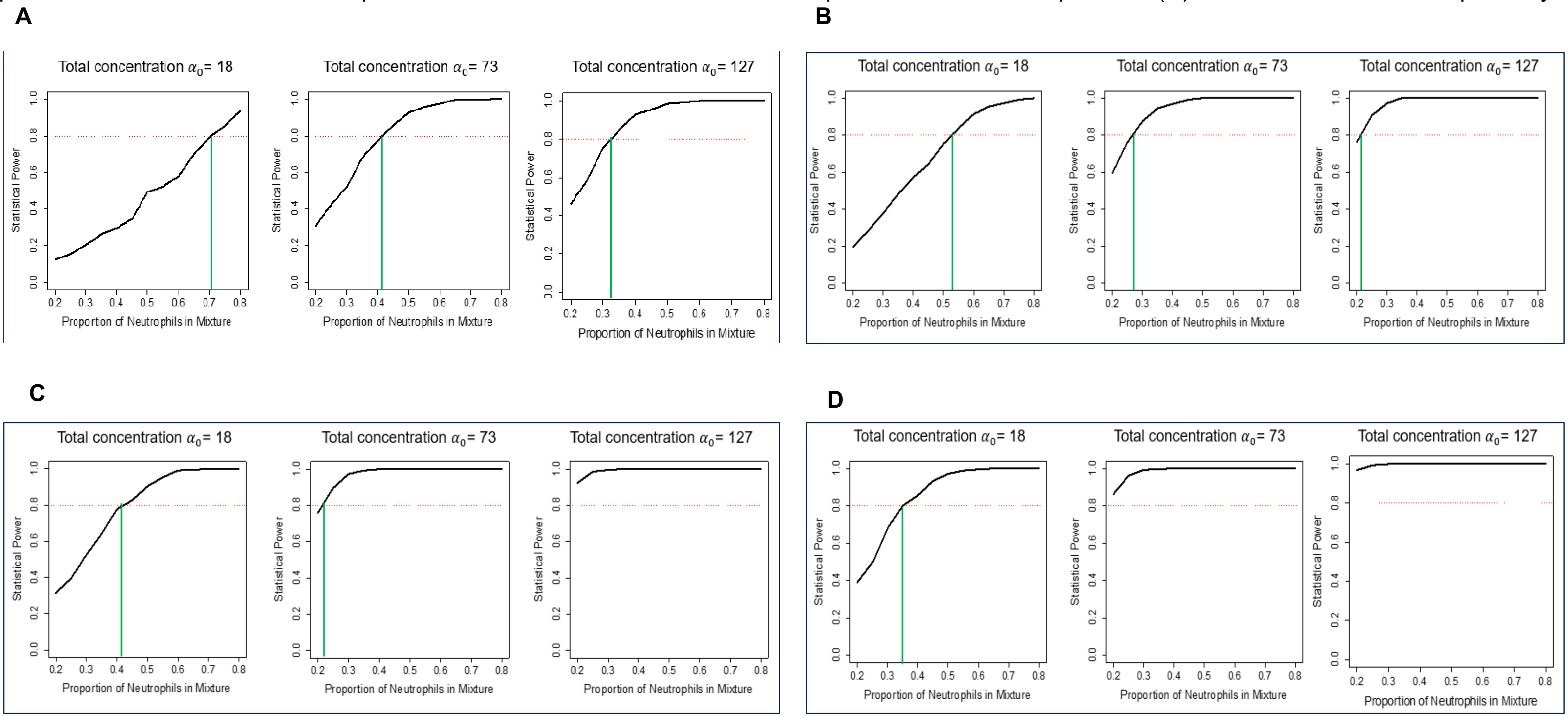

Supplementary Figure 6. Bland Altman analysis of FCM and methylation based deconvolution of CD4 T cell counts in 74 glioma subjects. Subjects included DEX exposed and non-exposed subjects and persons during and after chemoradiation. Source data are provided as a Source Data file.

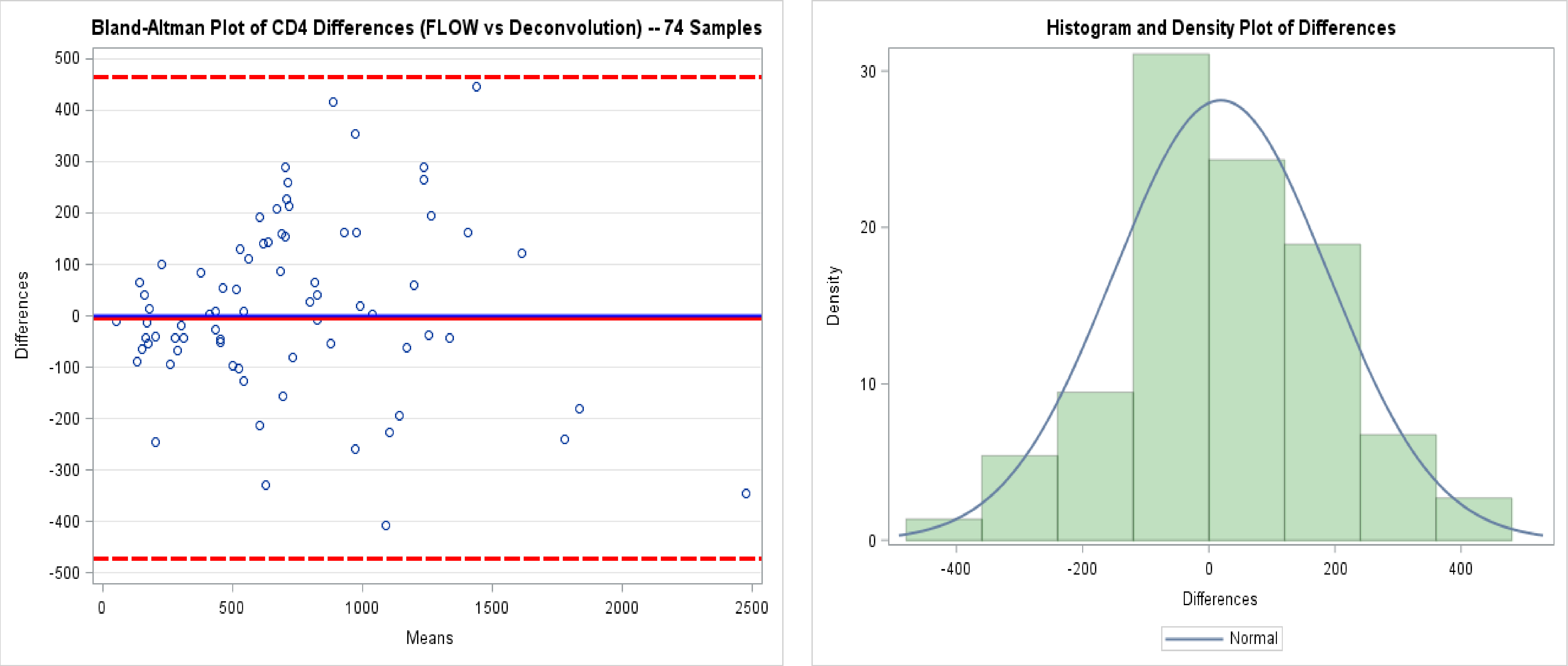

*The solid red line is the mean of the difference, dashed red lines are +/- 2 standard deviations of the difference.

Supplementary Figure 7: A series of boxplots showing the relationship between the Dirichlet concentration parameter on the variability of cellular proportions. The median and interquartile range are depicted within the box. The whiskers are the most extreme data points. Source data are provided as a Source Data file.

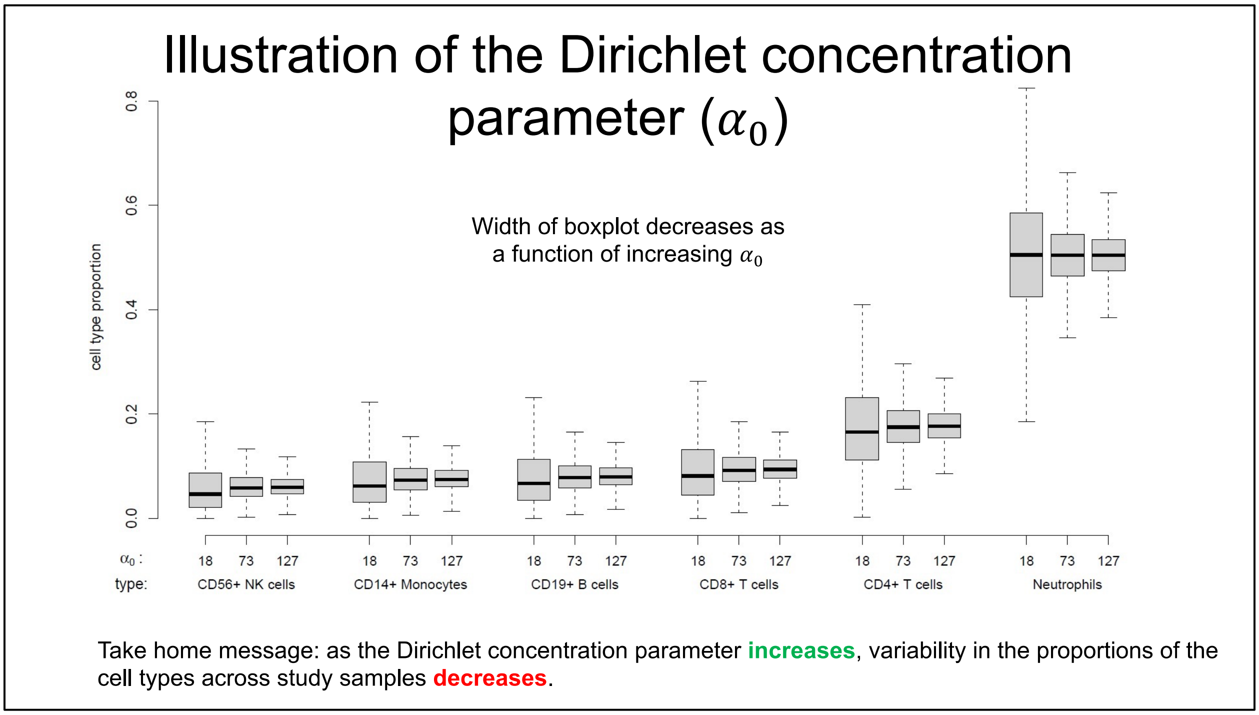

Supplementary Figure 8. FACS Gating Strategy

Forward and side scatter were employed to discriminate the non-doublet, non-debris, CD45+ leukocyte population. Further CD64+CD14+ gating identified the monocyte population. The HLA-DR neg/low gate in the monocyte population was set in each sample according to the HLA-DR neg/low granulocyte population in the same tube. These HLA-DR neg/low monocytes were further gated for CD16+/- expression. The CD16 gate was set in each sample according to the CD16- lymphocyte population in the same tube.

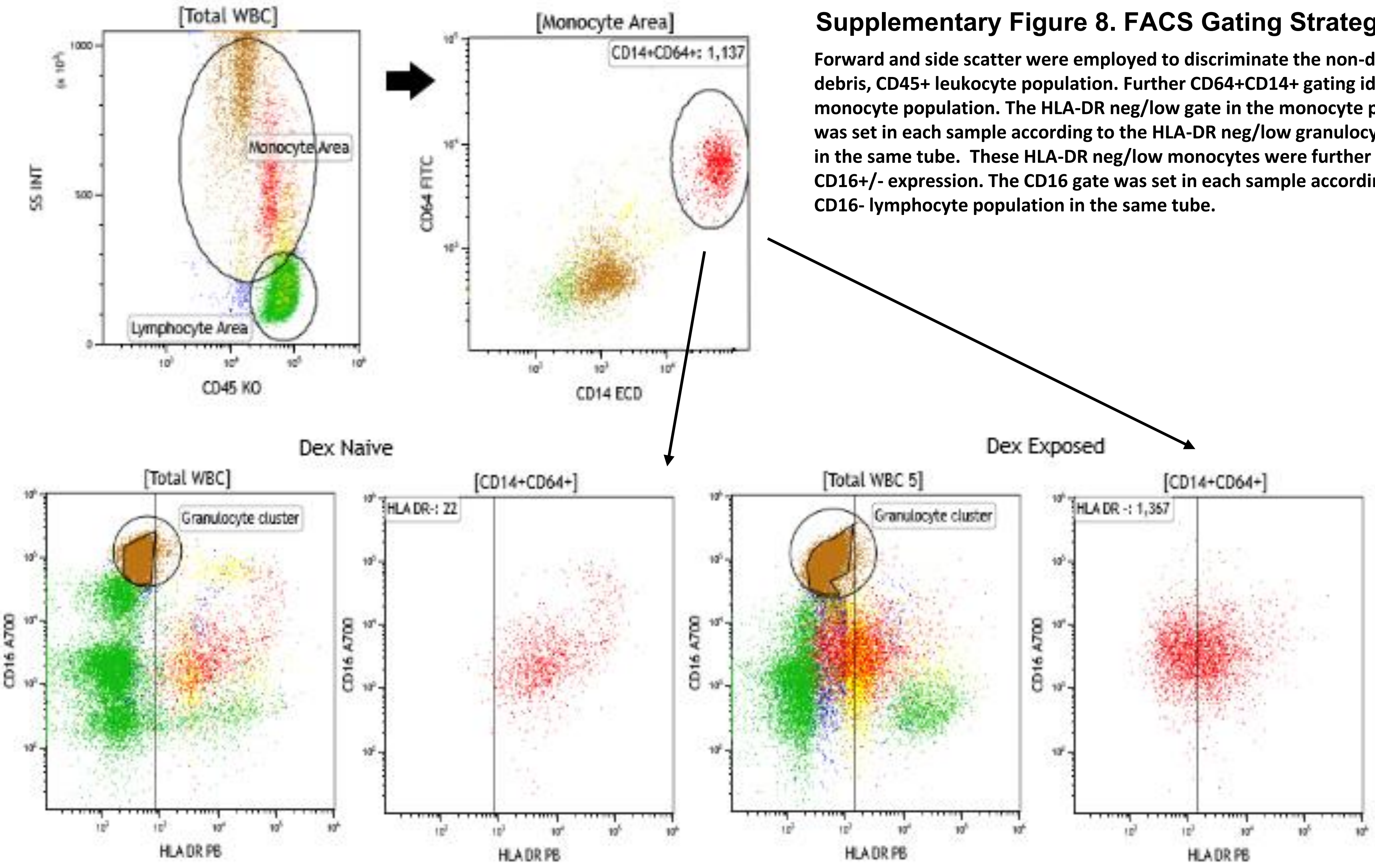

Supplementary Table 1. Demographic and Clinical Characteristics: Training Set of 135 Immune Profiles Study glioma patients, Test set of 99 AGS glioma patients, and 454 AGS controls (subjects without glioma)

Variables	IPS Glioma Patients (Training) (N=135)	AGS Glioma Patients (Test set) (N=99)	Subjects without glioma (Controls) (N=454[a])
Clinical and Demographic Variables			
Age at diagnosis (for glioma patients) or study enrollment (for controls), median (IQR)	51.0 (38.0, 62.0)	52.0 (45.0, 64.0)	52.0 (41.0, 63.0)
Dex-exposed[b]			
Missing	0	0	6
No	76 (56.3%)	0	447 (99.8%)
Yes	59 (43.7%)	99 (100%)	1 (0.2%)
Cumulative Dex Dose, median (IQR)	56.0 (24.5, 112.0)	NA	NA
Average Daily Dex Dose (mg/day)	8.0 (4.0, 12.0)	NA	NA
Sex			
Female	53 (39.3%)	39 (39.4%)	207 (45.6%)
Male	82 (60.7%)	60 (60.6%)	247 (54.4%)
Race			
Non-White	14 (10.4%)	19 (19.2%)	132 (29.1%)
White	121 (89.6%)	80 (80.8%)	322 (70.9%)
Vital Status			
Alive	107 (79.3%)	4 (4.0%)	NA
Dead	28 (20.7%)	95 (96.0%)	
WHO 2016 Glioma Classification			
IDH wild-type Glioblastoma	57 (42.2%)	80 (80.8%)	NA
IDH wild-type Astrocytoma	0	19 (19.2%)	
IDH mutant Glioblastoma	16 (11.9%)	0	
IDH mutant Astrocytoma	22 (16.3%)	0	
IDH mutant/1p19q co-deleted Oligodendroglioma	40 (29.6%)	0	
Glioma Diagnosis Grade			NA
Grade II	35 (25.9%)	6 (6.1%)	
Grade III	27 (20.0%)	13 (13.1%)	
Grade IV	73 (54.1%)	80 (80.8%)	
Chemotherapy at blood draw	NA		NA
Missing		3	
Current (0-7 days prior to draw)		17 (17.7%)	
None prior to blood draw		38 (39.6%)	
Prior (> 7 days before)		41 (42.7%)	
Radiation at blood draw	NA		NA
Missing		2	
Current (0-7 days prior to draw)		13 (13.4%)	
None prior to blood draw		25 (25.8%)	
Prior (> 7 days before)		59 (60.8%)	

Variables	IPS Glioma Patients (Training) (N=135)	AGS Glioma Patients (Test set) (N=99)	Subjects without glioma (Controls) (N=454[a])
Immune Cell Proportions, Ratios, and Counts			
Bcell proportions, median (IQR)	3.8 (2.6, 5.2)	1.9 (1.1, 2.7)	5.3 (4.0, 7.0)
Monocyte proportions, median (IQR)	6.5 (4.7, 8.4)	7.3 (5.1, 9.3)	7.4 (6.0, 9.4)
Neutrophil proportions, median (IQR)	66.8 (57.8, 81.7)	75.9 (66.8, 84.5)	57.9 (51.0, 65.5)
Natural Killer Cell proportions, median (IQR)	3.9 (2.6, 5.7)	2.5 (1.7, 4.1)	4.7 (3.4, 6.2)
$CD4^{+}T$ proportions, median (IQR)	10.0 (3.8, 16.1)	7.0 (3.1, 10.4)	15.7 (11.8, 19.7)
$CD8^{+}T$ proportions, median (IQR)	6.3 (3.0, 9.7)	4.9 (2.7, 7.8)	8.9 (6.1, 12.6)
$CD4^{+}T/CD8^{+}T$, median (IQR)	1.5 (1.0, 2.0)	1.5 (0.8, 2.5)	1.7 (1.2, 2.6)
LMR, median (IQR)	4.2 (2.7, 5.6)	2.3 (1.7, 3.5)	4.8 (3.8, 6.4)
NLR, median (IQR)	2.6 (1.6, 5.7)	4.6 (2.7, 7.9)	1.6 (1.2, 2.2)
Total Lymphocytes, median (IQR)	25.9 (14.2, 36.1)	16.7 (10.7, 25.4)	37.1 (29.4, 43.9)

[a]One control was dropped from the statistical models due to Dex exposure at blood draw.
[b]7 subjects were taking Dexamethasone within the previous 30 days.
Abbreviations: LMR: lymphocyte monocyte ratio; NLR: neutrophil lymphocyte ratio

Supplementary Table 2. FCM estimation of CD4 T cells and mMDSCs

Antibodies for FCM Detection of whole blood cells

Name ; Dilution*	Clone	Cat#	Vendor
CD45-KrO, ASR ; 1:19	J33	A96416	Beckman Coulter
**mMDSC -Myeloid Tube #2**			
CD11b-APC-A750, ASR ; 1:38	Bear1	A97052	Beckman Coulter
CD64-FITC, ASR ; 2:19	22	IM1604U	Beckman Coulter
CD14-ECD, ASR ; 1:19	RMO52	IM2707U	Beckman Coulter
HLA-DR-PB, ASR ; 1:38	Immu-357	A74781	Beckman Coulter
CD16-APC-A700, ASR ; 1:38	3G8	B20023	Beckman Coulter
**CD4+ T-Cells**			
CD3-APC-A750, ASR ; 1:40	UCHT1	A66329	Beckman Coulter
CD4-PC5.5, ASR ; 1:40	13B8.2	B16491	Beckman Coulter

*per 50uL whole blood, WBC not to exceed 1.0 x $10^{\wedge 7}$ cells per ml

Supplementary Table 3. Training dataset (N=135) blood leukocyte parameters and Dexamethasone status

Variable	No (N=76)	Yes (N=59)[b]	P-value[a]
NDMI Score, median (IQR)	-2.7 (-3.6, -1.9)	2.7 (1.5, 4.2)	< 0.001[a]
Non-cell specific methylation score, median (IQR)	-2.4 (-3.0, -1.7)	2.1 (1.2, 2.8)	< 0.001[a]
Leukocyte composition score, median (IQR)	-2.4 (-3.3, -1.2)	2.1 (0.9, 2.9)	< 0.001[a]
B cell proportions, median (IQR)	4.5 (3.3, 6.1)	2.7 (1.8, 3.9)	< 0.001[a]
Monocyte proportions, median (IQR)	7.3 (6.1, 9.1)	4.7 (3.0, 6.5)	< 0.001[a]
Neutrophil proportions, median (IQR)	58.7 (53.1, 64.2)	83.3 (75.8, 88.9)	< 0.001[a]
Natural Killer Cell proportions, median (IQR)	5.5 (4.2, 6.3)	2.6 (1.3, 3.5)	< 0.001[a]
CD4T proportions, median (IQR)	15.3 (11.3, 18.7)	3.5 (1.9, 6.9)	< 0.001[a]
CD8T proportions, median (IQR)	8.8 (6.2, 11.8)	2.9 (1.7, 5.4)	< 0.001[a]
CD4/CD8, median (IQR)	1.6 (1.3, 2.2)	1.3 (0.8, 1.7)	0.031[a]
Lymphocyte/monocyte ratio, median (IQR)	4.6 (3.8, 5.7)	2.9 (2.0, 4.5)	< 0.001[a]
Neutrophil/lymphocyte ratio, median (IQR)	1.7 (1.3, 2.3)	6.3 (3.7, 11.2)	< 0.001[a]
Total lymphocyte proportion, median (IQR)	34.9 (28.9, 40.2)	13.5 (7.9, 20.3)	< 0.001[a]
Absolute Lymphocyte Count (cells/ul), median (IQR)	2037 (1486, 2549)	1234 (899, 2014)	< 0.001[a]
Absolute CD8T Count (cells/ul), median (IQR)	505.5 (326.5, 661.5)	283.5 (187.8, 571.2)	0.018[a]
Absolute CD4T Count (cells/ul), median (IQR)	857.3 (606.5, 1100.2)	410.2 (199.1, 704.4)	< 0.001[a]
Absolute Bcell Count (cells/ul), median (IQR)	259.9 (194.7, 354.6)	296.6 (209.2, 425.3)	0.238[a]
Absolute Monocyte Count (cells/ul), median (IQR)	442.1 (355.8, 537.6)	521.8 (310.1, 771.8)	0.048[a]
Absolute NK Count (cells/ul), median (IQR)	316.8 (212.7, 417.4)	241.0 (187.2, 353.8)	0.157[a]
Absolute Neutrophil Count (cells/ul), median (IQR)	3306 (2364, 4570)	9162 (7001, 12172)	< 0.001[a]

a. Linear Model ANOVA
b. 52 subjects taking DEX at blood draw, 7 subjects within proceeding 30 days.

Abbreviations: IQR: Interquartile Range

Supplementary Table 4. Neutrophil specific loci CpG loci and gene characteristics, 28 NDMI CpG sites

Gene name (probe)	Function	Genomic Location Hg19	NR3C1 Binding Site
FKBP5 cg00052684	Chaperone protein and regulator of glucocorticoid receptor	Chr6:35694245; intron 1	YES<br>MYC, MAX, MAZ, EP300<br>Yes enhancer
CYTIP cg03915055	Regulation of b2-integrin signaling	Chr2:158301005; intron 4	YES<br>Enhancer/promoter
ZBTB16 cg25345365<br>ZBTB16 cg07375358	Transcriptional repressor POK<br>(POZ and Krüppel) proteins	Chr11:114050114; Intron 3<br>Chr11:114056431; intron 3	YES<br>NO Nearest site chr11:114050166<br>Yes enhancer
SLC2A1 cg02238943	Glucose transporter	Chr1:43401136; Intron 2	NO<br>NR3C1 993 bp<br>PAX5,IRF4, RELA, RUNX3,<br>Yes enhancer
BTBD12 cg02405193<br>BTBD12 cg27094376<br>(SLX4)	ATM substrate that functions as a scaffold for DNA repair activities	Chr16:3639839<br>Chr16:3639688<br>Exon 12	NO<br>MYC, MAX
SUPT3H cg02869300	Transcription initiation protein SPT3 homolog	Chr6:45042369<br>Intron 3	NO<br>Yes enhancer
RPTOR cg07786220	Scaffold for mTORC substrates<br>Cell growth, autophagy regulation	Chr17:78683082<br>Intron 4	NO<br>15kb from NR3C1
PLEK cg13060970	Protein kinase C signaling	Chr2:68592349 exon1	NO<br>MYC, MAX,MAZ,EP300<br>Promoter/enhancer
JARID2 cg13077031	Jumanji family transcriptional repressor	Chr6:15266782 intron 1	NO<br>IKZF1,MYC,MAX,MAZ<br>DNAse/FAIRE
FBXL14 cg13513622	Ubiquitination proteosomal degradation	Chr12:1702208 exon 1	NO<br>TF binding
ZC3H12D cg17478979	Regulates TLR signaling	Chr6:149772150, Exon 6	NO<br>DNAase Hyper EZH2 TF
RCAN3 cg18128887	Calcineurin inhibitor down-regulates NFAT-dependent cytokine gene expression	Chr1:24861708 Exon 5	NO<br>Enhancer, TF binding, DNAase, Flaire
PTPRM cg20071505	Regulates adhesion tyrosine phosphatase<br>Catenins cadherins	Chr18:8238684 intron 14	NO<br>Intron 14 TF binding FOXA1
NDRG1 cg20100745	Downregulates NFKB signaling	Chr8:134307728 intron 1	NO<br>TF binding site
RASA3 cg22104744	Ras GTPase-activating protein 3 modulates Th2 Th17 generation	Chr13:114829833 Intron 2	YES<br>MYC, MAX
EIF4G1 cg23189692	Translation initiation factor in immune response	Chr3:184050393 Intron 32	NO<br>TF binding site

Supplementary Table 5. Lists of explanatory variables in three DEX predictors

NDMI
CpGs
cg00052684
cg01994208
cg02405193
cg02869300
cg03915055
cg06216080
cg06769150
cg07375358
cg07786220
cg12230203
cg13060970
cg13077031
cg13121392
cg13513622
cg15416870
cg16729631
cg17478979
cg18128887
cg20071505
cg20100745
cg20799095
cg20932630
cg21195450
cg22104744
cg22520791
cg23189692
cg25345365
cg27094376

NonDMC
CpGs
cg00188443
cg00683922
cg01089474
cg02406277
cg02757819
cg02992366
cg03428193
cg03556491
cg03618348
cg04114389
cg04543901
cg04889800
cg05461268
cg05726764
cg05871802
cg05949667
cg06100756
cg06153925
cg06204030
cg06763776
cg07114727
cg07304483
cg07706850
cg08599355
cg09257039
cg09329516
cg10083098
cg10813029
cg10854441
cg10937743
cg10958808
cg11155735
cg11265221
cg11937033
cg12296597
cg12422154
cg13286857
cg13654097
cg13720639
cg13860849
cg13916733
cg14304236

NonDMC (continued)
CpGs
cg14712766
cg14727962
cg15379858
cg15467280
cg15564619
cg16634967
cg16915057
cg16982063
cg17218270
cg19197158
cg21058391
cg22023206
cg24740632
cg25310867
cg25899923
cg26408927
cg26651303
cg26737223
cg26958509

Cell Proportions
CD4T
Mono
Neu
NK
NLR

Supplementary Table 6. FACS isolation of putative mMDSCs

Antibodies for FACS Isolation of mMDSC Cells

Name ; Dilution Factor*	Clone	Cat#	Vendor
FITC anti-human CD3 Antibody ; 1:50	OKT3	317306	Biolegend
FITC anti-human CD56 (NCAM) Antibody ; 1:50	HCD56	318304	Biolegend
FITC anti-human CD19 Antibody ; 1:50	HIB19	302206	Biolegend
FITC PE anti-human CD14 Antibody ; 1:50	63D3	367104	Biolegend
PerCP/Cyanine5.5 anti-human CD33 Antibody ; 1:36	P67.6	366616	Biolegend
Brilliant Violet 421™ anti-human CD16 Antibody ; 1:50	B73.1	360724	Biolegend
Brilliant Violet 605™ anti-human CD11b Antibody ; 1:36	M1/70	101257	Biolegend
Biotin anti-human HLA-DR antibody 1:50	LN3	327004	Biolegend
CD15 Antibody, anti-human ; 1:50	VIMC6	130-114-008	Miltenyi Biotec

*6.0 x $10^{\wedge 3}$ washed cells per ul, pre-treated with 1:500 PE/Cyanine7 Streptavidin (Biolegend 405206)

Supplementary Table 7. Reproducibility of immune parameters: coefficients of variation of DEX predictors and 6 cell proportions

Parameter	Coefficient of variation (N=17 subjects)
NDMI	17.4%
Non-cell specific methylation score	24.5%
Leukocyte composition score	19.9%
Neutrophil proportion	1.1%
Monocyte proportion	6.1%
CD4 proportion	3.9%
CD8 proportion	2.7%
B cell proportion	10.0%
NK cell proportion	10.8%

Footnote: each subject sample run in replicate; 11/17 replicates were run in separate array batches and different arrays. CV estimated with root mean square method of Hyslop NP and White WH[1].

Supplementary Table 8. Associations of NDMI score with demographic and medication history in non-glioma controls (n=454)

Variable	Category	#	Mean NDMI	Median NDMI	St Dev	Min	Max	P-value**
Gender	Female	207	-3.27	-3.34	1.69	-8.27	3.67	0.68
	Male	247	-3.21	-3.21	1.61	-8.09	3.99	
Age Group	<40	107	-3.12	-3.25	1.75	-8.27	3.67	0.0002
	40-60	196	-3.58	-3.74	1.58	-7.69	3.35	
	60+	151	-2.87	-2.84	1.56	-7.78	3.99	
BMI Category	Missing	3	-3.91	-3.50	0.92	-4.97	-3.27	0.55
	<18.5	2	-3.18	-3.18	2.58	-5.01	-1.35	
	18.5 - 24.9	189	-3.35	-3.24	1.52	-8.09	1.11	
	25.0 - 29.9	155	-3.09	-3.14	1.67	-7.78	3.67	
	30+	105	-3.23	-3.33	1.83	-8.27	3.99	
Smoking Status	Missing	1	-4.97	-4.97	NA	-4.97	4.97	0.93
	Never Smoker	228	-3.25	-3.27	1.68	-8.27	3.67	
	Current Smoker	61	-3.27	-3.33	1.54	-7.03	0.31	
	Past Smoker	164	-3.20	-3.26	1.63	-7.78	3.99	
Race/Ethnicity	Asian	30	-3.03	-2.83	1.15	-5.12	-0.20	0.72
	Black	48	-3.41	-3.51	2.29	-7.78	3.99	
	Hispanic	46	-3.48	-3.69	1.41	-6.57	-0.56	
	Native American	2	-2.86	-2.86	3.15	-5.09	-0.64	
	Other	5	-3.17	-3.64	1.32	-4.78	-1.74	
	Pacific Islander	1	-5.13	-5.13	NA	-5.13	-5.13	
	White	322	-3.20	-3.21	1.60	-8.27	3.67	
BP Medications*	No	307	-3.28	-3.30	1.53	-8.27	3.67	0.04
	Yes	132	-2.94	-3.08	1.74	-7.78	3.99	
Any Corticosteroids*	No	412	-3.29	-3.30	1.49	-8.27	0.31	<0.001
	Yes	27	-1.45	-1.76	2.28	-4.89	3.99	
Corticosteroid Type*	Inhaled GC	9	-2.90	-2.54	1.00	-4.89	-1.76	0.01
	Oral Prednisone	18	-0.72	-1.11	2.42	-3.97	3.99	

Drug Category #1*<br>(All GC combined)	GC&BP	11	-0.91	-1.76	2.41	-3.41	3.99	<0.001
	No BP or GC	291	-3.36	-3.33	1.45	-8.27	0.31	
	Only BP	121	-3.12	-3.19	1.55	-7.78	0.19	
	Only GC	16	-1.82	-1.83	2.20	-4.89	3.67	
Drug Category #2*<br>(GC type separated)	No BP or GC	291	-3.36	-3.33	1.45	-8.27	0.31	<0.001
	Inhaled GC & BP	5	-2.43	-2.31	0.62	-3.41	-1.76	
	Only BP	121	-3.12	-3.19	1.55	-7.78	0.19	
	Only Inhaled GC	4	-3.50	-3.40	1.15	-4.89	-2.32	
	Only Oral GC	12	-1.27	-1.24	2.20	-3.97	3.67	
	Oral GC & BP	6	0.36	-0.61	2.65	-2.24	3.99	

* Reported at the time of blood draw. Medication information was available for n=439 controls (for 15 controls from AGS Series 1, medication categories were not abstracted so were not available to include).

** Linear model ANOVA.

Abbreviations: BP: blood pressure medication; GC: glucocorticoid

Supplementary Table 9. **Immune Profile Comparisons by Glucocorticoid Use at Blood Draw, Adult Glioma Study Controls**

Glucocorticoid use at blood draw	Neutrophil proportions	NLR	CD4 proportions	CD8 proportions	CD4/CD8 Ratio	NK cell proportions	B cell proportions	Monocyte proportions	Total Lymphocytes
No GC exposure (n=412), Median (IQR)	57.8 (51.1, 65.3)	1.6 (1.2, 2.2)	15.7 (12.0, 19.9)	8.8 (6.2, 12.5)	1.8 (1.2, 2.6)	4.7 (3.5, 6.2)	5.3 (4.0, 7.0)	7.4 (6.0, 9.3)	37.2 (29.5, 43.6)
Oral prednisone use (n=18), Median (IQR)	63.9 (59.1, 81.8)	2.1 (1.7, 5.3)	9.7 (6.4, 14.4)	8.2 (4.2, 11.3)	1.2 (1.0, 1.9)	3.9 (2.0, 5.2)	4.1 (3.0, 4.6)	7.1 (6.1, 9.8)	30.3 (15.6, 35.1)
Inhaled GC use (n=9), Median (IQR)	62.9 (60.8, 67.4)	2.1 (1.7, 2.2)	15.5 (13.6, 17.1)	4.9 (4.3, 12.5)	3.3 (1.2, 3.6)	3.2 (3.0, 4.5)	3.8 (3.0, 6.0)	7 (6.0, 8.2)	30.3 (29.4, 36.4)
P-value (Linear ANOVA comparing differences between all 3 groups)	< 0.001	< 0.001	< 0.001	0.56	0.28	0.08	0.04	0.85	< 0.001
Values in Prednisone exposed versus controls with no GC exposure	Increased	Increased	Decreased	Not Statistically Different	Not Statistically Different	Decreased	Decreased	Not Statistically Different	Decreased

Supplementary Table 10. Isolated Neutrophil NDMI and Whole Blood NDMI Scores for Samples from Dexamethasone Exposed Patients (see Figure 6e)

Sample	Timepoint	Isolated Neutrophil NDMI	Whole Blood NDMI	DEX at draw	Cumulative DEX dose	Ave Daily Dex Dose	Days on DEX	Histology
1 (post-surgery)	During Adj TMZ	3.198105781	-1.499171	Yes	29.5	1	30	GBM
2a (pre-surgery)*	Pre-surgery	6.267951205	4.648032	Yes	280	13.3	21	GBM
2b (post-surgery)*	Post-surgery	5.27063715	1.500488	Yes	101	6.3	16	GBM
3 (post-surgery)	Post-surgery	3.055926067	0.9477919	Yes	54	6.8	8	GBM
4 (pre-surgery)	Pre-surgery	5.586146291	3.795803	Yes	144	12	12	GBM

*Samples 2a and 2b were from the same patient at different timepoints (pre-surgery and post-surgery).

Supplementary Table 11. Cox multivariate model of NDMI and glioma survival in 74 UCSF Adult Glioma Study patients with extent of resection (EOR) and tumor volume available (both EOR and pre-operative tumor volume added to the model)

Variable	Hazard Ratio	P-value*
NDMI Score	1.60	$9.6x10^{-4}$
Age (yrs, continuous)	1.08	$8.1x10^{-4}$
World Health Organization 2016 Classification		
IDH WT GBM (n=26)	3.40	0.12
Oligodendroglioma (n=40)	0.18	0.02
Astrocytoma Gr 2/3 (baseline, n=8)	1.0	NA
CD4 T-cells (proportions)	1.07	0.14
BMI (basal metabolic index)	1.01	0.82
Race (white vs non-white)	0.93	0.89
Dexamethasone use at blood draw	0.67	0.35
Extent of tumor resection	0.98	0.19
Pre-operative tumor volume	1.01	0.03
Tumor Location		
Overlapping Sites	3.57	0.07
Parietal Lobe	0.67	0.41
Frontal/Cerebrum/Temporal/Other (baseline)	1.0	NA

* P-values were calculated using the Wald Test.

Supplementary Table 12. Cox multivariate model of NDMI and glioma survival in 74 UCSF Adult Glioma Study patients with extent of resection (EOR) and tumor volume available (only EOR added to the model)

Variable	Hazard Ratio	P-value*
NDMI Score	1.42	$6.7x10^{-3}$
Age (yrs, continuous)	1.08	$2.8x10^{-4}$
<u>World Health Organization 2016 Classification</u>		
IDH WT GBM (n=26)	2.44	0.19
Oligodendroglioma (n=40)	0.17	0.01
Astrocytoma Gr 2/3 (baseline, n=8)	1.0	NA
CD4 T-cells (proportions)	1.04	0.32
BMI (basal metabolic index)	1.01	0.86
Race (white vs non-white)	1.19	0.74
Dexamethasone use at blood draw	0.65	0.30
Extent of tumor resection	0.97	0.005
<u>Tumor Location</u>		
Overlapping Sites	4.35	0.03
Parietal Lobe	0.69	0.45
Frontal/Cerebrum/Temporal/Other (baseline)	1.0	NA

* P-values were calculated using the Wald Test.

Supplementary Table 13. Antibodies for FACS Isolation of Neutrophils

Antibodies for FACS Isolation of Neutrophils

Name	low density dilution factor*	high density dilution factor*	Clone	Cat#	Vendor
FITC anti-human CD3 Antibody	1:840	1:210	OKT3	317306	Biolegend
FITC anti-human CD56 (NCAM) Antibody	1:560	1:140	HCD56	318304	Biolegend
FITC anti-human CD49b- Antibody	1:560	1:140	9F10	304316	Biolegend
APC/Cyanine7 anti-human CD19-	1:315	1:63	HIB19	302218	Biolegend
Alexa Flour 488 anti-human CD123 Antibody	1:560	1:140	TS2/16	306036	Biolegend
PerCP/Cyanine5.5 anti-human CD33 Antibody	1:210	1:35	P67.6	366616	Biolegend
CyLight 550 CEACAM8/CD66b Antibody	1:315	1:63	6/40C	NBP2-54627R	Novus Biologicals

*per 1.0 x 10$^{\wedge 7}$ low density granulocytes & 5.0 x 10$^{\wedge 7}$ high density granulocytes

Supplementary References:

1. Hyslop NP, White WH: Estimating precision using duplicate measurements. J Air Waste Manag Assoc 59:1032-9, 2009
