# Supplementary material for: DNA methylation as a pharmacodynamic marker of glucocorticoid response and glioma survival
Source: Nat Commun. 2022 Sep 20;13:5505. doi: 10.1038/s41467-022-33215-x (PMC9486797; doi:10.1038/s41467-022-33215-x)
Supplement: Supplementary file 3 — Description of Additional Supplementary Files [file 41467_2022_33215_MOESM3_ESM.pdf]

Supplementary Data 1: List of 2621 CellDMC neutrophil CpG loci

Supplementary Data 2: Transcription factor binding at 28 NDMI CpG sites by probe
