# Supplementary material for: DNA methylation as a pharmacodynamic marker of glucocorticoid response and glioma survival
Source: Nat Commun. 2022 Sep 20;13:5505. doi: 10.1038/s41467-022-33215-x (PMC9486797; doi:10.1038/s41467-022-33215-x)
Supplement: Supplementary file 6 — Reporting Summary [file 41467_2022_33215_MOESM6_ESM.pdf]

Corresponding author(s): Wiencke, John
Last updated by author(s): Aug 26, 2022

Reporting Summary

Nature Portfolio wishes to improve the reproducibility of the work that we publish. This form provides structure for consistency and transparency in reporting. For further information on Nature Portfolio policies, see our Editorial Policies and the Editorial Policy Checklist.

Statistics

For all statistical analyses, confirm that the following items are present in the figure legend, table legend, main text, or Methods section.

n/a	Confirmed	
☐	☒	The exact sample size (n) for each experimental group/condition, given as a discrete number and unit of measurement
☐	☒	A statement on whether measurements were taken from distinct samples or whether the same sample was measured repeatedly
☐	☒	The statistical test(s) used AND whether they are one- or two-sided<br>*Only common tests should be described solely by name; describe more complex techniques in the Methods section.*
☐	☒	A description of all covariates tested
☐	☒	A description of any assumptions or corrections, such as tests of normality and adjustment for multiple comparisons
☐	☒	A full description of the statistical parameters including central tendency (e.g. means) or other basic estimates (e.g. regression coefficient) AND variation (e.g. standard deviation) or associated estimates of uncertainty (e.g. confidence intervals)
☐	☒	For null hypothesis testing, the test statistic (e.g. F, t, r) with confidence intervals, effect sizes, degrees of freedom and P value noted<br>*Give P values as exact values whenever suitable.*
☒	☐	For Bayesian analysis, information on the choice of priors and Markov chain Monte Carlo settings
☒	☐	For hierarchical and complex designs, identification of the appropriate level for tests and full reporting of outcomes
☒	☐	Estimates of effect sizes (e.g. Cohen's d, Pearson's r), indicating how they were calculated

Our web collection on statistics for biologists contains articles on many of the points above.

Software and code

Policy information about availability of computer code

Data collection	Custom code was not used to collect data for this study, but we created R Shiny code (see below) to generate NDMI scores.
Data analysis	The online application we developed using the R Shiny application to calculate neutrophil dexamethasone methylation index (NDMI) scores from methylation data can be found at the following URL: https://btcdb.shinyapps.io/ndmi/

For manuscripts utilizing custom algorithms or software that are central to the research but not yet described in published literature, software must be made available to editors and reviewers. We strongly encourage code deposition in a community repository (e.g. GitHub). See the Nature Portfolio guidelines for submitting code & software for further information.

Data

Policy information about availability of data

All manuscripts must include a data availability statement. This statement should provide the following information, where applicable:

- Accession codes, unique identifiers, or web links for publicly available datasets
- A description of any restrictions on data availability
- For clinical datasets or third party data, please ensure that the statement adheres to our policy

Data Availability: Methylation and phenotype data used in this manuscript are available through dbGaP controlled access. Methylation and phenotype data from the Adult Glioma Study are available through dbGaP Study Accession phs001497.v2.p1 (https://www.ncbi.nlm.nih.gov/projects/gap/cgi-bin/study.cgi?study_id=phs001497.v2.p1). Methylation and phenotype data from the Immune Profiles Study are available through dbGaP Study Accession phs002998.v1.p1 (https://www.ncbi.nlm.nih.gov/projects/gap/cgi-bin/study.cgi?study_id=phs002998.v1.p1). Source data files have been provided with this manuscript. Please note that we do not have IRB approval to release individual level data for 15 out of 457 AGS controls (from Series 1), so these controls are not included in the source files.

Field-specific reporting

Please select the one below that is the best fit for your research. If you are not sure, read the appropriate sections before making your selection.

☒ Life sciences ☐ Behavioural & social sciences ☐ Ecological, evolutionary & environmental sciences

For a reference copy of the document with all sections, see nature.com/documents/nr-reporting-summary-flat.pdf

Life sciences study design

All studies must disclose on these points even when the disclosure is negative.

Sample size	The training set sample size was determined by selecting all Immune Profiles Study patients who had a pre-surgery sample run on the 850k methylation array (n=135 glioma patients).<br>The test set was determined by selecting Adult Glioma Study patients (n=99) who were exposed to Dexamethasone at blood draw, were IDH-WT, TERT-WT, and 1p19q intact, and had 850K methylation data available. All available controls who had methylation array data available (n=454) were also selected for the test set.<br>The survival evaluation set was determined by selecting the remaining Adult Glioma Study patients (n=429) who had Dexamethasone use information at blood draw and methylation data available.
Data exclusions	Only adults 18 and over were included in the study. For the Immune Profiles Study, patients were excluded from this analysis if they did not have a pre-surgery sample run on the EPIC array. For the Adult Glioma Study test and survival set, cases and controls without a blood sample run on the 850k methyation array were excluded. Some analyses excluded NDMI outliers.
Replication	A test dataset was used to validate findings in the training set. The test set included glioma patients and controls recruited from a separate study. Our replication attempts were successful.
Randomization	Samples were randomized within plates for methylation arrays. Since these studies were not clinical trials, randomization was not used for subject selection/eligibility.
Blinding	We did not use blinding in this study because this is not a clinical trial. However, lab analyses and deconvolution measures were completed without any knowledge of patient case status or clinical factors.

Reporting for specific materials, systems and methods

We require information from authors about some types of materials, experimental systems and methods used in many studies. Here, indicate whether each material, system or method listed is relevant to your study. If you are not sure if a list item applies to your research, read the appropriate section before selecting a response.

Materials & experimental systems

n/a	Involved in the study	
☐	☒	Antibodies
☒	☐	Eukaryotic cell lines
☒	☐	Palaeontology and archaeology
☒	☐	Animals and other organisms
☐	☒	Human research participants
☒	☐	Clinical data
☒	☐	Dual use research of concern

Methods

n/a	Involved in the study	
☒	☐	ChIP-seq
☐	☒	Flow cytometry
☒	☐	MRI-based neuroimaging

Antibodies

Antibodies used	FCM estimation of CD4 T cells and mMDSCs: Antibodies used include: CD45-KrO, ASR (Clone:J33, Cat#: A96416, Vendor: Beckman Coulter);CD11b-APC-A750, ASR (Clone: Bear1, Cat#: A97052, Vendor: Beckman Coulter);CD64-FITC, ASR (Clone: 22, Cat#: IM1604U, Vendor: Beckman Coulter);CD14-ECD, ASR (Clone:ClRMO52, Cat#:IM2707U, Vendor: Beckman Coulter);HLA-DR-PB, ASR (Clone:Immu-357, Cat#: A74781, Vendor: Beckman Coulter);CD16-APC-A700, ASR (Clone:3G8, Cat#: B20023, Vendor: Beckman Coulter);CD3-APC-A750, ASR (Clone:UCHT1, Cat#: A66329, Vendor: Beckman Coulter);CD4-PC5.5, ASR (Clone:13B8.2, Cat#: B16491, Vendor: Beckman Coulter).<br>FACS isolation of putative mMDSCs: Antibodies used include:;FITC anti-human CD3 Antibody (Clone:13B8.2, Cat#B16491 Vendor: Beckman Coulter);FITC anti-human CD56 (NCAM) Antibody (Clone:HCD56, Cat# 318304 Vendor:Biolegend); APC/Cyanine7 anti-human CD19 Antibody (Clone:HIB19 Cat#302218 Vendor:Biolegend); PE anti-human CD14 Antibody (Clone:63D3, Cat#367104 Vendor:Biolegend); PerCP/Cyanine5.5 anti-human CD33 Antibody (Clone:P67.6, Cat#366616 Vendor:Biolegend); Brilliant Violet 421TM anti-human CD16 Antibody(Clone:B73.1, Cat#360724 Vendor:Biolegend); Brilliant Violet 605TM anti-human CD11b Antibody (Clone:M1/70, Cat#101257 Vendor:Biolegend); Biotin anti-human HLA-DR antibody(Clone:LN3, Cat#327004 Vendor:Biolegend); CD15 Antibody, anti-human(Clone:VIMC6, Cat#130-114-008 Vendor:Miltenyi Biotec).<br>FACS isoloation of neutrophils: Antibodies used include FITC anti-human CD3 Antibody (Clone: OKT3, Cat#317306,

Vendor:Biolegend), FITC anti-human CD56 (NCAM) Antibody (Clone: HCD56, Cat#318304, Vendor:Biolegend), FITC anti-human CD49b - Antibody (Clone:9F10, Cat#304316, Vendor:Biolegend), APC/Cyanine7 anti-human CD19- (Clone: HIB19, Cat#302218, Vendor:Biolegend), Alexa Flour 488 anti-human CD123 antibody (Clone: TS2/16, Cat#306036, Vendor:Biolegend), PerCP/Cyanine5.5 anti-human CD33 antibody (Clone: P67.6, Cat#366616, Vendor:Biolegend), CyLight 550 CEACAM8/CD66b antibody (Clone: 60/40C, Cat#NBP2-54627R, Vendor:Novus Biologicals).

Validation

Antibodies used

CD3-FITC (BioLegend Cat# 317306, RRID:AB_571907); CD56-FITC (BioLegend Cat# 318304, RRID:AB_604100); CD49b-FITC (BioLegend Cat# 304316, RRID:AB_2561759); CD19-APC/Cyanine7 (BioLegend Cat# 302218, RRID:AB_314248); CD123-Alexa Flour 488 (BioLegend Cat# 306036, RRID:AB_2629570); CD33-PerCP/Cyanine5.5 (BioLegend Cat# 366616, RRID:AB_2566418); CEACAM8/CD66b-cyLight 550 Mouse Novus biologicals 1:40 (FC/FACS) Cat# NBP2-54627R ; CD45-KO (Beckman Coulter Cat# A96416, RRID:AB_2888654); CD11b-APC-A750 (Beckman Coulter Cat# A97052, RRID:AB_2800450); CD64-FITC (Beckman coulter Cat# IM1604U); CD14-ECD (Beckman Coulter Cat# IM2707U, RRID:AB_130853); HLA-DR-PB (Beckman Coulter Cat# A74781, RRID:AB_2892134); CD16-APC-A700 Mouse Beckman Coulter 1:19 FC Cat# B20023; CD3-APC-A750 (Beckman Coulter Cat# A66329, RRID:AB_2876783); CD4-PC5.5 (Beckman Coulter Cat# B16491); CD3-FITC (BioLegend Cat# 317306, RRID:AB_571907); CD56-FITC (BioLegend Cat# 318304, RRID:AB_604100); CD19 APC/Cyanine7 (BioLegend Cat# 302218, RRID:AB_314248); CD14-PE (BioLegend Cat# 367104, RRID:AB_2565888); CD33 PerCP/Cyanine5.5 (BioLegend Cat# 366616, RRID:AB_2566418); CD16-Brilliant Violet 421 (BioLegend Cat# 360724, RRID:AB_2616914); CD11b Brillant Violet 605 (BioLegend Cat# 101257, RRID:AB_2565431); HLA-DR-Biotin (BioLegend Cat# 327004, RRID:AB_893580); CD15-APC (Miltenyi Biotec Cat# 130-114-008, RRID:AB_2733338);

Validation:

CD3-FITC (BioLegend Cat# 317306, RRID:AB_571907) http://antibodyregistry.org/AB_571907;
CD56-FITC (BioLegend Cat# 318304, RRID:AB_604100) http://antibodyregistry.org/AB_604100;
CD49b-FITC (BioLegend Cat# 304316, RRID:AB_2561759) http://antibodyregistry.org/AB_2561759;
CD19-APC/Cyanine7 (BioLegend Cat# 302218, RRID:AB_314248) http://antibodyregistry.org/AB_314248;
CD123-Alexa Flour 488 (BioLegend Cat# 306036, RRID:AB_2629570) http://antibodyregistry.org/AB_2629570;
CD33-PerCP/Cyanine5.5 (BioLegend Cat# 366616, RRID:AB_2566418) http://antibodyregistry.org/AB_2566418 ;
CEACAM8/CD66b-cyLight 550 (Novus biologicals, Cat# NBP2-54627R) Reactivity: Human. Applications: ELIZA, FC/FACS, IHC, WB; Antibody was purchased from vendor and validated based on vendors quality control requirements.
CD45-KO (Beckman Coulter Cat# A96416, RRID:AB_2888654) http://antibodyregistry.org/AB_2888654 ;
CD11b-APC-A750 (Beckman Coulter Cat# A97052, RRID:AB_2800450) http://antibodyregistry.org/AB_2800450 ;
CD64-FITC (Beckman coulter Cat# IM1604U) Reactivity: Human. Cross Reactivity: Indian Rhesus. Applications: FC/FACS. 7 references on www.citeab.com.
CD14-ECD (Beckman Coulter Cat# IM2707U, RRID:AB_130853) http://antibodyregistry.org/AB_130853;
HLA-DR-PB (Beckman Coulter Cat# A74781, RRID:AB_2892134) http://antibodyregistry.org/AB_2892134;
CD16-APC-A700 (Beckman Coulter, Cat# B20023) Antibody was purchased from vendor and validated based on vendors quality control requirements.
CD3-APC-A750 (Beckman Coulter Cat# A66329, RRID:AB_2876783) http://antibodyregistry.org/AB_2876783;
CD4-PC5.5 (Beckman Coulter Cat# B16491) Reactivity: Human; Cross Reactivity: Hamadyras Baboon, Olive Baboon. Applications: FC/FACS. 6 references on www.citeab.com.
CD3-FITC (BioLegend Cat# 317306, RRID:AB_571907) http://antibodyregistry.org/AB_571907;
CD56-FITC (BioLegend Cat# 318304, RRID:AB_604100) http://antibodyregistry.org/AB_604100 ;
CD19 APC/Cyanine7 (BioLegend Cat# 302218, RRID:AB_314248) http://antibodyregistry.org/AB_314248 ;
CD14-PE (BioLegend Cat# 367104, RRID:AB_2565888) http://antibodyregistry.org/AB_2565888 ;
CD33 PerCP/Cyanine5.5 (BioLegend Cat# 366616, RRID:AB_2566418) http://antibodyregistry.org/AB_2566418 ;
CD16-Brilliant Violet 421 (BioLegend Cat# 360724, RRID:AB_2616914) http://antibodyregistry.org/AB_2616914 ;
CD11b Brillant Violet 605 (BioLegend Cat# 101257, RRID:AB_2565431) http://antibodyregistry.org/AB_2565431 ;
HLA-DR-Biotin (BioLegend Cat# 327004, RRID:AB_893580) http://antibodyregistry.org/AB_893580 ;
CD15-APC (Miltenyi Biotec Cat# 130-114-008, RRID:AB_2733338) http://antibodyregistry.org/AB_2733338

Human research participants

Policy information about studies involving human research participants

Population characteristics	Human research participants included adult glioma patients enrolled in the UCSF Immune Profiles Study and adult glioma patients and controls enrolled in the UCSF Adult Glioma Study. The UCSF Immune Profiles Study (IPS) is a prospective neuro-oncology clinic-based collection of blood samples, imaging, and other clinical data from adult glioma patients (recruitment started in 2018). The Adult Glioma Study was a case-control study of glioma patients newly diagnosed between 1991 and 2012 who were residents of the SF Bay Area or patients of the UCSF Neuro-oncology clinic and age, race/ethnicity, and gender-matched controls without any history of glioma recruited through random digit dialing or from the UCSF blood draw lab. Patients in both studies were 18 years of age and older (median age of cases and controls was ~52 years). For cases in the training and test sets, 61% were male and 39% were female. For controls in the test set, 54% were male and 46% were female.
Recruitment	For the Immune Profiles Study, eligible adult glioma patients were recruited through the UCSF Neurosurgery (NS) and Neuro-Oncology services prior to their surgery and written consent is obtained. For the Adult Glioma Study, adult glioma patients newly diagnosed between 1991 and 2012 who were residents of the SF Bay Area or patients of the UCSF Neuro-oncology clinic were recruited in-person, by mail, or by phone. Age, race/ethnicity, and gender-matched controls without any history of glioma were recruited through random digit dialing or from the UCSF blood draw lab. For the Adult Glioma Study, we tried to limit selection bias by using population-based recruitment for cases (in addition to clinic recruitment) and random digit dialing for controls for Series 1-4. We also matched controls to cases by age, race/ethnicity, and gender. Controls recruited in the final series of AGS (Series 5) were recruited through the UCSF blood draw lab and not through population-based methods. For the Immune Profiles Study, we tried to limit selection bias by using multiple sources to identify eligible glioma patients receiving surgery at UCSF and documenting patients who are missed, ineligible, or refuse.
Ethics oversight	UCSF Human Research Protection Program Institutional Review Board approved these studies. Informed consent was obtained from all participants.

Note that full information on the approval of the study protocol must also be provided in the manuscript.

Flow Cytometry

Plots

Confirm that:

☒ The axis labels state the marker and fluorochrome used (e.g. CD4-FITC).

☒ The axis scales are clearly visible. Include numbers along axes only for bottom left plot of group (a 'group' is an analysis of identical markers).

☒ All plots are contour plots with outliers or pseudocolor plots.

☒ A numerical value for number of cells or percentage (with statistics) is provided.

Methodology

Sample preparation	Blood samples (3 ml EDTA preserved whole blood) were collected, and total white blood cell (WBC) counts were recorded. Following red blood cell lysis, cells were mixed with a monoclonal antibody mixture including CD45, CD64, CD16, CD11b, HLA-DR in a single tube, incubated in the dark for 20 minutes, washed, and fixed with 0.1% formaldehyde prior to acquisition on the Navios EX Flow Cytometer (Beckman Coulter Life Sciences).
Instrument	Navios EX Flow Cytometer (Beckman Coulter Life Sciences)
Software	Data were analyzed using Kaluza Analysis Software 2.1 (Beckman Coulter Life Sciences).
Cell population abundance	The purity of flow cytometry sorted cells was confirmed with an independent method using specific DNA methylation deconvolution, the results indicated the purity of samples used for specific analyses.
Gating strategy	Forward and side scatter were employed to discriminate the non-doublet, non-debris, CD45+ leukocyte population and cells counted. Further CD64+CD14+ gating identified the monocyte population. The HLA-DR neg/low gate in the monocyte population was set in each sample according to the HLA-DR neg/low granulocyte population in the same tube. These HLA-DR neg/low monocytes were further gated for CD16+/- expression. The proportion of each cell type present in the sample was then calculated based on the number of CD45+ leukocytes. The mMDSC proportion of CD45+ leukocytes counted in the acquisition tube was calculated. Total WBC counts were then used to calculate the final cell/ul concentrations of mMDSCs in the blood. Antibodies were used to stain CD45, CD3, and CD4 to identify the CD4 T cells. Within the CD45 population, the low side scatter intensity CD3+ cluster identified the CD3+ population. These CD3+ cells were further gated for CD4+/- expression. The CD4 T-cell proportion of CD45+ leukocytes counted in the acquisition tube was calculated. Total WBC counts were then used to calculate the final cell/ul concentrations of CD4 T-cells in the blood.

☒ Tick this box to confirm that a figure exemplifying the gating strategy is provided in the Supplementary Information.
